# Serious Drug-Drug Interactions in the Prescriptions of Diabetic Patients

**DOI:** 10.3390/medsci3040093

**Published:** 2015-10-01

**Authors:** Veintramuthu Sankar, Yousif Saaed, Rinku Mary Joseph, Hudson Azizi, Pinky Mariyam Thomas

**Affiliations:** Department of Pharmaceutics, PSG College of Pharmacy, Coimbatore, Tamilnadu-641004, India; E-Mails: eswari1005@gmail.com (Y.S.); rinkumj0890@gmail.com (R.M.J.); vallima@rediffmail.com (H.A.); pinkypinks1410@gmail.com (P.M.T.)

**Keywords:** diabetes mellitus, drug interactions, identification, prescriptions

## Abstract

A large number of drugs are introduced every year, and newer interactions between medications are increasingly reported. Clinically significant drug interactions can occur when two or more drugs are taken in combination. With the continuing increase in the list of drugs capable of interactions, detection of these interactions from prescriptions becomes more important to ensure effective patient care. The aim of this study is to identify the possible drug interactions in the prescriptions of diabetic inpatients and to make physicians aware of these interactions to prevent the occurrence of clinically adverse effects. In a specially designed and validated data entry format, data for the following criteria were collected: drugs prescribed, major drug class prescribed, pharmacological classification of the observed drug interaction, and frequently occurring drug interactions. All the possible drug interactions were identified and evaluated using standard drug interaction reference books and databases. During the study period, 50 prescriptions of diabetic inpatients were screened randomly. Out of these prescriptions, 35 (70%) prescriptions had at least one possible drug-drug interaction***.*** The major classes of drugs causing interactions included cardiac drugs (92%), analgesic drugs (66%), antibiotic drugs (52%), antidiabetic drugs (26%), diuretic drugs (26%), and antipsychotic drugs (24%). This study showed that 34 (68%) of the above prescriptions had minor interactions, 33(66%) had moderate interactions, and 10 (20%) had severe interactions. Of these, the drugs prescribed specifically for diabetes caused only nine moderate interactions. Thus, screening of prescriptions by the clinical pharmacist will help to minimize clinical occurrence of major/severe drug interactions in diabetic patients.

## 1. Introduction

Drug interactions are a daily challenge for physicians and screening all potential interactions in the prescriptions has become very cumbersome and virtually impossible [1]. Most drugs have multiple pharmacologic effects in patients, especially the newer and more complex drugs that are marketed currently. Clinically significant drug interactions can occur when two or more drugs are taken in combination [2]. Drug-drug interaction is one cause of adverse reactions leading to an increase in risk of hospitalization and added health care costs. With the continuing increase in the list of drugs capable of interactions, detection of these interactions from prescriptions becomes more important to ensure effective patient care [3].

In recent years, serious drug interactions with some widely used drugs have emerged. We need to reevaluate the screening procedures for potential drug interactions and ensure that preventable drug interactions are identified and information regarding the same is passed on to healthcare professionals [4].

A drug interaction can be defined as a measurable modification (in magnitude or duration) of the action of one drug by prior or concomitant administration of another substance (including prescription and non-prescription drugs, food or alcohol) [5]. Drug interactions are believed to occur in 3% to 7% of patients taking up to 10 medications, and in as many as 20% patients taking 10 to 20 medications. It is estimated that drug interactions cause up to 2.8% of all hospitalizations [6].

All this information prompted us to come up with a simple method for finding potential drug interactions. The aim of this study is to identify possible serious drug interactions in the prescriptions of diabetic inpatients and make physicians aware of them so as to prevent occurrence/reoccurrence of clinical adverse events.

## 2. Materials and Methods

### 2.1. Study Site and Study Population 

The study was conducted at a 950-bed, multi-specialty hospital in Coimbatore, India. Patients diagnosed with DM and admitted to the general medicine ward were included in the study. Intensive care patients were excluded from the study as their treatment pattern is different and cannot be generalized to all population categories.

### 2.2. Study Design

The study was carried out for a period of 45 days from 16 April 2012 to 31 May 2012. It was a prospective observational interventional study.

Data collection: A data entry form for collecting patient details was prepared. It included patient details like name, age, sex, weight, inpatient number, date of admission, date of discharge, reason for admission, past medical history, past medication history, social history, drugs prescribed, dose, frequency, duration of therapy, route of administration, category of drugs, diagnosis, and drug interactions.

Screening: The collected data were thoroughly screened for drug interactions with the help of trusted/standard websites like Medscape and drugs.com, as well as standard reference textbooks for drug interactions. The identified drug interactions were documented and analyzed [7,8,9].

The patients diagnosed with DM were selected randomly. The randomization was done by blindly selecting the diabetic patient’s inpatient code numbers used in the hospital. The protocol of the study was approved by the Institutional Human Ethics Committee of the hospital. Informed consent was obtained from all participants prior to collecting data. Statistical analysis of the data collected was done using SPSS version 19 (IBM Corp, Armonk, NY, USA). 

## 3. Results

### 3.1. Total Number of Prescriptions Screened in Diabetic Patients

During the study period, a total of 50 prescriptions of diabetic inpatients were screened. Out of these, 35 (70%) prescriptions had at least one possible drug-drug interaction (Table 1). 

**Table 1:** Total number of prescriptions screened in diabetic patients.

SI. No.	Category of Prescription Screened	Number of Prescriptions	Percentage (%)
1.	Prescription with drug interactions	35	70%
2.	Prescription without drug interactions	15	30%

### 3.2. Age Distribution of Diabetic Patients Studied

**Figure 1:**
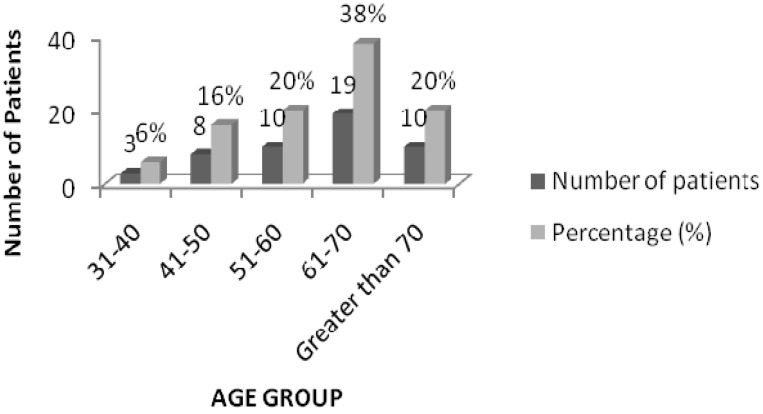
Age distribution of patients with drug interactions.

The results of the age categorization revealed that maximum drug interactions (38%) were found in the prescriptions of patients in the age group 61–70 years (19 patients), followed by 10 patients (20%) each in the 51–60 and above 70 age groups (Figure 1).

### 3.3. Number of Drugs Prescribed in Diabetic Prescriptions

The number of drugs prescribed were 3–5 in five prescriptions (10%), 6–10 drugs in 27 (54%), 11–15 drugs in 13 (26%) and more than 15 drugs in five prescriptions (10%) (Table 2).

**Table 2:** Number of drugs prescribed in DM prescriptions.

Number of Drugs	Number of Prescription	Percentage (%)
3–5	5	10
6–10	27	54
11–15	13	26
More than 15	5	10

### 3.4. Major Classes of Drugs Prescribed in Diabetic Prescriptions

The major classes of drugs prescribed were insulin/OHA in 50 prescriptions (100%), proton pump inhibitors in 37 (74%), NSAIDs in 26 (52%), antibiotics in 26 (52%), antihypertensives in 25 (50%), multivitamins and minerals in 18 (36%), antihyperlipidemics in 15 (30%), serotonin antagonist in 13 (26%), bronchodilators in 12 (24%), antiplatelets in 12 (24%), antihistamine in 7 (14%), anticoagulants in seven (14%) and benzodiazepines in five prescriptions (10%) (Table 3).

**Table 3:** Major classes of drugs prescribed in DM prescriptions.

Drug Class	No. of Prescription	Percentage (%)
Antibiotics	26	52
Anticoagulants	7	14
Antigout	3	6
Antihistamines	7	14
Antihyperlipidemic	15	30
Antihypertensive	25	50
Antiplatelets	12	24
Benzodiazepine	5	10
Bronchodilators	12	24
Diuretics	4	8
Insulin	26	52
Multivitamin & minerals	18	36
NSAIDs	26	52
OHA	24	48
Opioid analgesic	11	22
Proton pump inhibitor	37	74
Serotonin antagonist	13	26
Steroid	5	10
Thyroid hormone	7	14

### 3.5. Number of Drug Interactions per Prescription in Diabetic Prescriptions 

A study on the number of drug interactions per prescription indicated that at least one drug interaction was observed in two prescriptions (4%), followed by two drug interactions in four prescriptions (8%), three interactions in two prescriptions (4%),and more than four interactions in 27 (54%) prescriptions [10] (Table 4).

**Table 4:** Number of drug interactions per prescription in DM prescriptions (*N* = 35).

Number of Interaction	Number of prescription	Percentage (%)
1	2	4
2	4	8
3	2	4
4 and above	27	54

### 3.6. Major Classes of Drugs Causing Drug Interaction in Diabetic Prescriptions 

The major classes of drugs causing drug interactions were cardiac drugs (92%), analgesic drugs (66%), antibiotic drugs (52%), anti diabetic drugs (26%), diuretic drugs (26%), and antipsychotic drugs (24%) (Table 5).

**Table 5:** Major classes of drugs causing interactions in DM prescriptions.

Classes of Drugs	Number of Times	Percentage (%)
ACE inhibitors	6	12
Alpha blockers	1	2
Aminoglycoside	1	2
Analgesic	33	66
Angiotensin receptor blockers	8	16
Antibiotics	26	52
Anticoagulants	9	18
Antidiabetic drugs	13	26
Antiepileptic drugs	3	6
Antihyperlipidemic	11	22
Antiplatelet drugs	11	22
Antipsychotic drugs	12	24
Beta blockers	11	22
Bronchodilators	10	20
Calcium channel blockers	11	22
Carbamazepines	6	12
Cardiac drugs	46	92
Cardiac glycoside	9	18
Cephalosporin	4	8
Diuretics	13	26
Fluoroquinolones	6	12
Insulin	4	8
Macrolide	2	4
OHA	9	18
Others	5	10
Penicillin	8	16
Proton pump inhibitors	8	16
Serotonin antagonist	7	14
Steroids	7	14
Vitamins & Minerals	11	22

### 3.7. Individual Drug in Each Class Causing Interaction

On an individual drug basis the data revealed that Aspirin (40%), Clopidogrel (16%), Digoxin (14%), and Levofloxacin (12%) were the major interacting drugs in the selected prescriptions (Table 6).

**Table 6:** Major drugs interacting in DM prescriptions.

Drugs	Number of Times	Percentage (%)
Amoxicillin	4	8
Aspirin	20	40
Atorvastatin	5	10
Clopidogrel	8	16
Digoxin	7	14
Enoxaparin	5	10
Heparin	5	10
Insulin	4	8
Levofloxacin	6	12
Nifedipine	5	10
OHA	5	10
Pantoprazole	4	8

### 3.8. Classification of Drug Interaction Found in Prescriptions of Diabetic Patients

**Figure 2:**
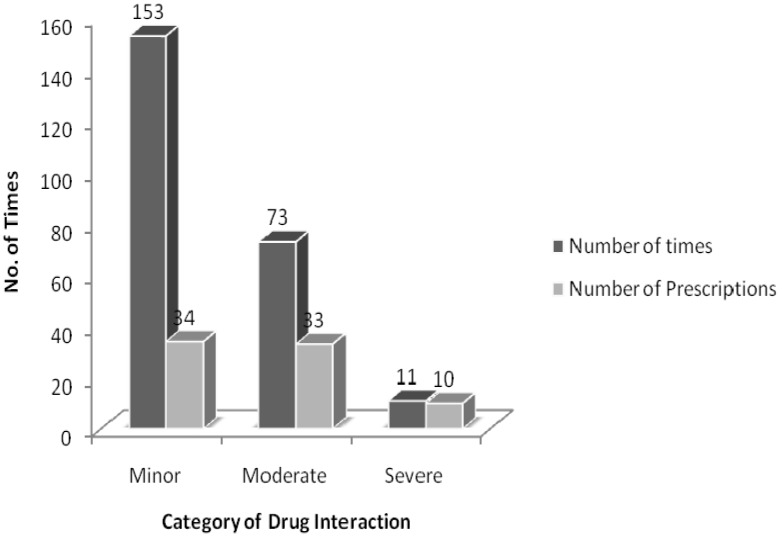
Classification of drug interactions in the prescriptions of diabetic patients.

The drug interactions were classified based on their respective pharmacological effects. In our study, 34 prescriptions (68%) had minor interactions, 33 prescriptions (66%) had moderate interactions, and 10 prescriptions (20%) had severe interactions (Figure 2). Regarding the drugs prescribed specifically for DM, only nine moderate interactions were observed (Table 7). None of the serious interactions were caused by these same drugs. All the serious interactions observed were caused by aspirin. The serious interactions identified in the screened prescriptions and their management is reported in Table 8.

**Table 7:** Drug interactions identified with antidiabetic drugs (moderate interactions).

S.No	Drugs	No. of Times	Effect	Alternative/Management
1.	Glibenclamide + diclofenac	1	Increased effect of glibenclamide.	Use with caution. Monitor blood sugar.
2.	Glibenclamide + ranitidine	1	Increased effect of glibenclamide.	Use with caution. Monitor blood sugar.
3.	Glibenclamide + hydrocortisone	1	Reduced effects of glibenclamide.	Use with caution. Monitor blood sugar.
4.	Glimipride + budesonide	1	Budesonide reduces the effect of glimipride.	Use with caution. Monitor blood sugar.
5.	Glimipride + aspirin	1	Increased effect of glimipride.	Use with caution. Monitor blood sugar.
6.	Metformin + budesonide	1	Reduced effect of metformin.	Use with caution. Monitor blood sugar.
7.	Insulin + aspirin	2	Increased effect of insulin.	Use with caution. Monitor blood sugar.
8.	Insulin + levofloxacin	1	Levofloxacin disturbs blood glucose hemostasis.	Monitor blood sugar closely.
9.	Insulin + metoprolol	1	Increased effect of insulin.	Use with caution. Monitor blood sugar.

**Table 8:** Serious interactions identified in the screened prescriptions.

S.No	Drugs	No. of Times	Effect	Alternative/Management
1	Aspirin + Ketorolac	2	Aspirin causes additive toxicity with Ketorolac.	Avoid combination.
2	Aspirin + Enoxaparin	1	May potentiate the risk of bleeding.	It should be undertaken with caution. Close clinical and laboratory observation for bleeding complications is recommended.
3	Enoxaparin + Aspirin	2	Enoxaparin has an additive effect with aspirin.	Monitor hematological parameters. Use combination with extreme caution.
4	Aspirin +Lornoxicam	1	Aspirin causes additive toxicity with Lornoxicam.	Avoid combination.
5	Aspirin + Diclofenac	1	Aspirin causes additive toxicity with Diclofenac.	Avoid combination.
6	Aspirin + Piroxicam	2	Aspirin causes additive toxicity with Piroxicam.	Avoid combination.
7	Aspirin + Heparin	2	Aspirin has an additive effect with heparin.	Use combination with extreme caution. Monitor hematological parameters.

## 4. Discussion

Diabetic patients often have a number of coexisting health problems. Therefore, in addition to anti-diabetic drugs, other drugs are often needed to control these problems. In our study, 70% of the screened prescriptions had at least one drug interaction and 54% of prescriptions had more than four drug interactions. The drug combinations identified in this study that have the potential to cause serious drug interactions (aspirin + lornoxicam, aspirin + diclofenac, aspirin + piroxicam) should be avoided in all hospitals. 

Through this study, 61 moderate interactions and eight serious interactions were reported. Aspirin and clopidogrel interacted three times more than the other drugs and hence should be administered with caution. All the identified significant drug interactions should be monitored for physiological, hematological, and pharmacological effects by the clinical pharmacist.

Most of these interactions were caused by drugs used to treat co-morbidities that occur along with DM. It is estimated from previous studies that people over 65 take an average of seven drugs at any one time to treat a variety of illnesses [11]. Similar results were obtained in our study, which showed that 54% of the prescriptions included 6–10 drugs and most of these were those concerning patients above 60 years. Maximum interactions (38%) were found in the 61–70 age group. The results are comparable with the study by Kohler GI (2000), where the patients who experienced drug interactions had a mean age of 64.8 ± 9.7 years. The reason for this could be that, with advancing age, the levels of plasma protein (mainly albumin) availability go down and there is also an imbalance in enzyme function [10,11,12]. A change in the drug dosage regimen is required to prevent potential drug interactions in patients of this age group [12]. In general, the literature reveals that as the number of drugs and age increases, the probability of drug interactions also increases [12,13].

The most common co morbidities that occurred for the DM patients included infections, followed by hypertension and dyslipidemia. This information was obtained from the prescriptions, as 52% of the prescriptions included antibiotics, 50% included antihypertensives, and 30% included lipid-lowering agents. In previous studies, the most common co morbidity identified was hypertension, followed by dyslipidemia and diabetic complications (microvascular and macrovascular) [10,12].

In our study, the major class of drug that caused interaction was cardiac drugs (92%) and NSAIDs (66%), followed by antibiotics (52%). In previous studies, potential interactions occurred between glucose-lowering agents and other drugs. Relevant pharmacological agents were those that are widely co administered in diabetic patients, such as antihypertensives and lipid-lowering agents [12,14]. Studies also reported that most of the drug reactions are caused by a small group of commonly prescribed NSAIDS, anticoagulants, and antibiotics [5,10,11]. Similar results were obtained in our study.

However, there was several limitations to our study. Our study was based on the screening of only a small number of prescriptions. By screening a bigger population subgroup, the results could be more generalized to all categories of the population. Also, we failed to differentiate between clinically significant and non-significant drug interactions. In future studies, we need to identify and differentiate between pharmacological and clinical drug interactions in order to prevent the occurrence of serious adverse events.

Minimizing the risk of drug interactions should be one of the most important goals in drug therapy because interactions can result in significant morbidity and mortality [7,8]. Health care providers should take responsibility for the safe prescribing of medication by avoiding potential drug interactions that can result in adverse reactions [5,9].

## 5. Conclusions

Potential drug interactions can be predicted and dealt with if there is close teamwork between physicians and pharmacists immediately after the medication is prescribed.

The most appropriate approach to avoiding drug interactions is for a hospital pharmacist to screen the total medication chart of every individual patient. A database system that could detect drug interactions in the prescriptions at the level of online prescribing or billing or dispensing would be a significant advancement to check and minimize serious drug interactions.

The risk of drug interactions increases exponentially with the number of drugs given to a patient. Without a well-designed, computerized drug interaction monitoring system or a proper database, the investigation of incidences of drug interactions in prescriptions in hospitals is limited and the consequential development of a comprehensive surveillance program for drug interactions in hospitals is not feasible. 

An updated database system on drug interactions monitored by a pharmacist in hospitals could ensure rational drug therapy and effective patient care, and prevent the occurrence of serious and clinically significant adverse events.
